# Mapping Solute Clearance From the Mouse Hippocampus Using a 3D Imaging Cryomicrotome

**DOI:** 10.3389/fnins.2021.631325

**Published:** 2021-03-22

**Authors:** Daphne M. P. Naessens, Johannes G. G. Dobbe, Judith de Vos, Ed VanBavel, Erik N. T. P. Bakker

**Affiliations:** ^1^Department of Biomedical Engineering and Physics, Amsterdam UMC, Amsterdam Neuroscience, University of Amsterdam, Amsterdam, Netherlands; ^2^Department of Biomedical Engineering and Physics, Amsterdam UMC, Amsterdam Movement Sciences, University of Amsterdam, Amsterdam, Netherlands

**Keywords:** cerebrospinal fluid, hippocampus, interstitial fluid, solute clearance, subarachnoid space, 3D imaging cryomicrotome

## Abstract

The hippocampus is susceptible to protein aggregation in neurodegenerative diseases such as Alzheimer’s disease. This protein accumulation is partially attributed to an impaired clearance; however, the removal pathways for fluids and waste products are not fully understood. The aim of this study was therefore to map the clearance pathways from the mouse brain. A mixture of two fluorescently labeled tracers with different molecular weights was infused into the hippocampus. A small subset of mice (*n* = 3) was sacrificed directly after an infusion period of 10 min to determine dispersion of the tracer due to the infusion, while another group was sacrificed after spreading of the tracers for an additional 80 min (*n* = 7). Upon sacrifice, mice were frozen and sectioned as a whole by the use of a custom-built automated imaging cryomicrotome. Detailed 3D reconstructions were created to map the tracer spreading. We observed that tracers distributed over the hippocampus and entered adjacent brain structures, such as the cortex and cerebroventricular system. An important clearance pathway was found along the ventral part of the hippocampus and its bordering interpeduncular cistern. From there, tracers left the brain via the subarachnoid spaces in the directions of both the nose and the spinal cord. Although both tracers followed the same route, the small tracer distributed further, implying a major role for diffusion in addition to convection. Taken together, these results reveal an important clearance pathway of solutes from the hippocampus.

## Introduction

Several neurodegenerative diseases are characterized by the accumulation and aggregation of proteins. These include aggregates of amyloid-β in Alzheimer’s disease, α-synuclein in Parkinson’s disease, and huntingtin in Huntington’s disease (Ross and Poirier, 2004). Even though these neurodegenerative diseases exhibit differences in clinical manifestation and the anatomical brain regions that are affected, the process of degeneration associated with protein aggregation appears to be remarkably similar (Davis et al., 2018; Soto and Pritzkow, 2018). Multiple studies hypothesized that these accumulations stem from a disrupted balance between the production and clearance of these proteins. The traditional view on waste clearance is drainage of interstitial fluid (ISF) into the cerebrospinal fluid (CSF), in combination with removal across the blood–brain barrier (Tarasoff-Conway et al., 2015). However, a study by Iliff and colleagues challenged this view and proposed an additional clearance route via the so-called glymphatic system. In this pathway, subarachnoid CSF enters the brain parenchyma via perivascular spaces along penetrating arteries, where it mixes with the ISF. From there, the fluid disperses toward the perivenous spaces and leaves the brain interstitium into the subarachnoid space, a process that depends on glial cells (Iliff et al., 2012). Once in this compartment, solutes and CSF can exit the skull through arachnoid villi or along perineural routes through the foramina in the skull that drain into the extracranial lymphatic vessels, as shown in mice (Ma et al., 2017).

The extent to which specific anatomical brain regions use particular clearance pathways is not completely clear. In previous work by our group, fluorescently labeled tracers were infused into the striatum of mice to investigate the clearance route from this brain structure (Bedussi et al., 2015). The majority of the tracers drained into the adjacent lateral ventricle, from where they dispersed along the cisterns on the ventral side of the brain. Subsequent clearance of the large molecular weight tracer was observed across the cribriform plate, which is in agreement with the observations in the study of Ma et al. (2017).

In the present study, we aimed to outline the clearance pathways from the hippocampus of mice. This anatomical brain structure is known to be susceptible to protein aggregation of particularly amyloid-β, and is therefore one of the most affected brain regions in Alzheimer’s disease (Braak and Braak, 1991; Selkoe, 2002; Ross and Poirier, 2004). Since injection of tracers in the brain may affect physiological parameters, such as the intracranial pressure (ICP), we minimized the quantity and infusion rate of the tracer solution. Yet, tracer infusion inevitably leads to dispersion of the tracer due to the infusion itself. For this reason, we sacrificed a subset of animals directly after infusion to reveal the experimentally induced spreading of the tracer. The majority of animals were sacrificed after an additional spreading period that followed the tracer infusion, which aimed to examine dispersion through physiological mechanisms. Whole mice, with an intact cranium, were subsequently sectioned in an imaging cryomicrotome, and tracer distribution was assessed using the resulting 3D reconstructions.

## Materials and Methods

### Animals

In this study, a total of 11 male mice were used. These mice were randomly assigned to two different experimental groups: one group of three mice that were sacrificed directly after infusion of the fluorescent tracers, and one group of eight mice that were sacrificed after an additional spreading period after tracer infusion. One mouse died during the experiment, leaving seven mice in this experimental group. The C57BL/6JOlaHsd mice were obtained from Harlan (Netherlands) at 12 weeks of age. They were kept until 39.7 ± 0.1 weeks of age for the experimental procedure. The animals were housed in groups on a 12-h light/12-h dark schedule and had free access to standard laboratory food and water. All experiments were approved by the Academic Medical Center Animal Ethics Committee, and were conducted in accordance with the ARRIVE guidelines and European Union guidelines for the welfare of laboratory animals (Directive 2010/63/EU).

### Chemicals and Reagents

Two different fluorescently labeled dextrans were used as tracers to study the clearance pathways from the mouse hippocampus. Fluorescein labeled dextran (D7136, 500 kDa, Ex 494 nm/Em 512 nm) and Texas Red labeled dextran (D3328, 3 kDa, Ex 595 nm/Em 615 nm) were purchased from Molecular Probes, and were dissolved in artificial CSF (aCSF—135 mM NaCl, 5.4 mM KCl, 1 mM MgCl_2_, 1.8 mM CaCl_2_, 5 mM HEPES, pH 7.4) to a final concentration of 30 mg/mL.

### Surgical Procedure

Animals were weighed prior to the experimental procedure (36.1 ± 1.4 g, *n* = 10). The surgical procedure was performed under isoflurane inhalation anesthesia (Pharmachemie B.V.) and was kept between 2.0 and 3.0% in O_2_ at a delivery rate of 2.0 L/min. After induction of the general anesthesia, the core body temperature was measured with a rectal probe and was maintained at 36–37°C using a heating pad. Ophthalmic ointment (Duratears^®^, Alcon) was applied to prevent dehydration of the eyes. Mice were turned in the prone position, and the head was immobilized in a stereotaxic frame (Stoelting). The scalp was shaved, and a longitudinal incision was made to expose the skull. The stereotaxic coordinates of the CA1 region of the hippocampus were determined according to the Paxinos and Franklin mouse brain atlas (second edition, 2001) as 2.0 mm caudal from bregma, 1.6 mm lateral from the sagittal suture, and 1.5 mm ventral from the skull surface. 10% xylocaine (AstraZeneca B.V.) was applied as additional local anesthesia, and a small hole was drilled using a dental drill. A 34-gauge needle (Hamilton) was inserted, which was connected to a polythene catheter and syringe. Subsequently, 1.0 μL of the dextran mixture was infused at a controlled flow rate of 0.1 μL/min using a syringe pump (Harvard Apparatus). A subset of mice was sacrificed directly after infusion of the tracer mixture (10 min). The other group of animals was kept anesthetized for an additional 80 min to allow the tracer mixture to spread through the brain, while the needle was kept in place. These time points were based on data from a previous study by our group (Bedussi et al., 2015). Prior to sacrifice, heparin (LEO Pharma) was injected into the bloodstream via the dorsal penile vein and was allowed to circulate for 2 min. Animals were then sacrificed with an overdose of Euthasol^®^ (AST Farma B.V.) that was injected intravenously. All mice were subsequently transcardially perfused with phosphate-buffered saline (PBS). The teeth and skin were removed in order to protect the knife and prevent artifacts that may develop during the sectioning and imaging procedures in the imaging cryomicrotome. Animals were then snap-frozen in liquid nitrogen as a whole and stored at −20°C until further use.

### 3D Cryomicrotome Imaging

Whole mice were embedded in a cylindrical holder in 3.0% carboxymethylcellulose sodium solvent (Sigma) mixed with 0.1% black ink (VWR), and were frozen at −20°C for at least 24 h. 3D optical imaging was performed using a custom-built automated imaging cryomicrotome (Spaan et al., 2005; van den Wijngaard et al., 2013). All samples were serially cut from the thorax to the nose in slices of 45 μm. After each slice, the surface of the remaining tissue was imaged using a 4096 × 4096 pixels, 16-bit cooled charged-coupled device (CCD) camera (Apogee Alta U-16) equipped with an adjustable focus lens (Nikon 70-180 mm). Images of the fluorescent tracers were acquired using LED illumination in combination with narrow-band excitation and emission filters. Fluorescein labeled dextran was visualized at Ex 480 nm (bandwidth 20 nm)/Em 535 nm (bandwidth 50 nm) for 6000 ms, while Ex 577 nm (bandwidth 20 nm)/Em 635 nm (bandwidth 30 nm) for 8000 ms was used for the Texas Red labeled dextran. Reflection images were also obtained to visualize the contours of the sample at Ex 577 nm (bandwidth 20 nm)/Em 577 nm (bandwidth 30 nm) for 500 ms. All images were acquired using 1 × 1 binning, resulting in an in-plane resolution of 15 μm.

### Image Processing and Analysis

Sequential images were pre-processed using a program written in LabVIEW (National Instruments, Austin, TX, United States). Artifacts due to stuck pixels were eliminated by assigning these pixels the median value of their neighbors. Images were scaled to an isotropic voxel size of 45 μm. In-house developed software was subsequently used to stack the sequential images, to segment the distribution pattern of the tracers, and to create 3D polygon meshes representing the tracer spreading. For segmentation, a fixed intensity threshold was set for each fluorescent tracer, excluding all voxels with an intensity below the threshold value. The threshold was determined by examining the intensity values in regions where the tracer was absent, e.g., bone and muscle tissue, and was set at a level that ensured tracer was not segmented due to tissue autofluorescence. Intensity thresholds of the individual tracers were kept constant between all mice.

The total dispersion volumes of the different tracers were calculated by adding up the number of voxels with intensities above the threshold and multiplying this count by the voxel volume. Next to this, two observers (DN and EB) assessed the tracer distribution by scoring either the presence or absence of the fluorescent dextrans in a number of different anatomical structures. This was examined in all individual slices of the total imaged volume of each mouse. Both observers were blinded to the experimental group the mice were allocated.

### Statistical Analysis

A generalized estimating equation (GEE) model was used to estimate the effect size of the experimental procedure on the dispersion of the two different fluorescent tracers through the central nervous system. The presence of fluorescence signal of the individual tracers was used as the dependent variable. Odds ratios (OR) with 95% confidence intervals (CI) and their corresponding *p*-values are given. All other data are reported as mean ± SEM. In Figure 3, the Mann–Whitney *U* test was performed to compare the dispersion volumes of the fluorescent tracers. Differences between the two experimental groups were considered significant if *p* ≤ 0.05. Statistical analyses were performed in IBM SPSS Statistics (version 26), and GraphPad Prism Software (version 8.0.2) was used to visualize the data.

## Results

### 3D Reconstruction of the Tracer Dispersion Patterns

To outline the clearance pathways from the hippocampus in mice, we infused fluorescently labeled dextrans as tracer molecules and assessed their dispersion through the body. The tracer mixture consisted of two dextrans with different conjugated fluorophores and molecular weights. The low molecular weight, Texas Red labeled tracer has a comparable weight to that of the amyloid-β protein and should therefore reflect the removal pathway via the ISF of this and other endogenous proteins and solutes of a similar size. We also included a high molecular weight fluorescein labeled tracer, which we anticipated to show relatively little dispersion via diffusion, but rather relies on bulk flow.

**FIGURE 1:**
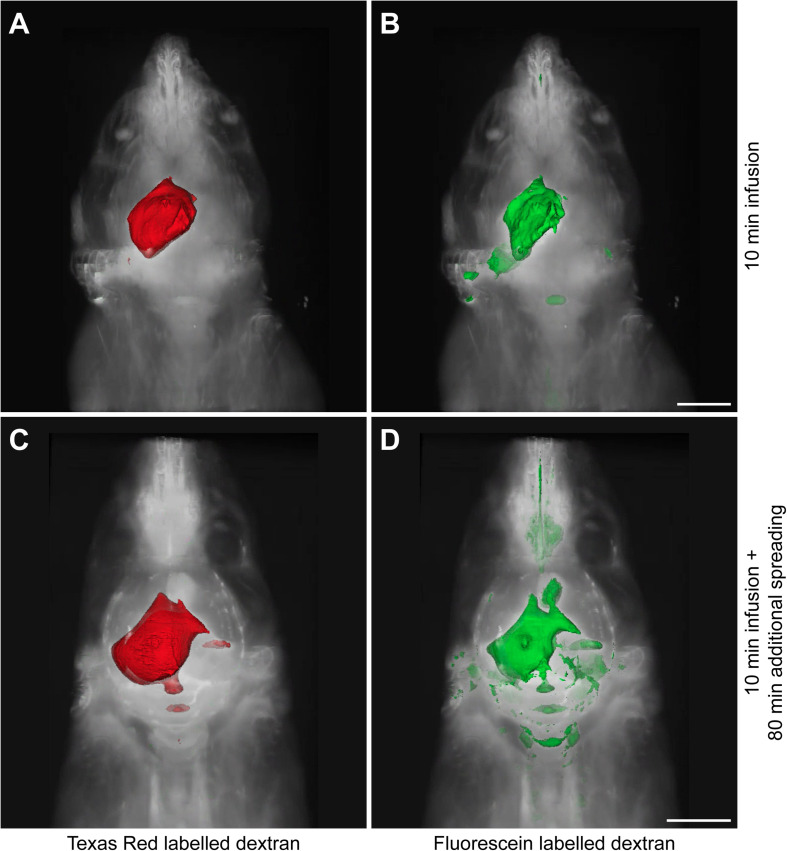
Representative 3D reconstructions of the tracer distribution through the body. The polygon meshes delineate regions above a fixed intensity threshold that was kept constant for each fluorescent tracer. **(A,B)** The dispersion of Texas Red labeled and fluorescein labeled dextrans after the infusion period of 10 min. **(C,D)** The spreading pattern of the same fluorescent tracers after an additional spreading period of 80 min that followed the infusion period of 10 min. Scale bar represents 5000 μm.

Since the distribution of these tracers may be affected by the infusion itself, we sacrificed the animals at two different time points. A small subset of mice (*n* = 3) was sacrificed directly after tracer infusion, while another group (*n* = 7) was kept anesthetized for an additional 80 min to allow further dispersion of the tracers trough physiological mechanisms. Figure 1 shows representative 3D reconstructions of the tracer dispersion both after infusion and the additional spreading period. The 3D reconstructions of the dispersion of the fluorescein and Texas Red labeled dextrans for the individual mice are shown in Supplementary Figures 1, 2, respectively.

### Tracer Dispersion to Different Anatomical Structures

**FIGURE 2:**
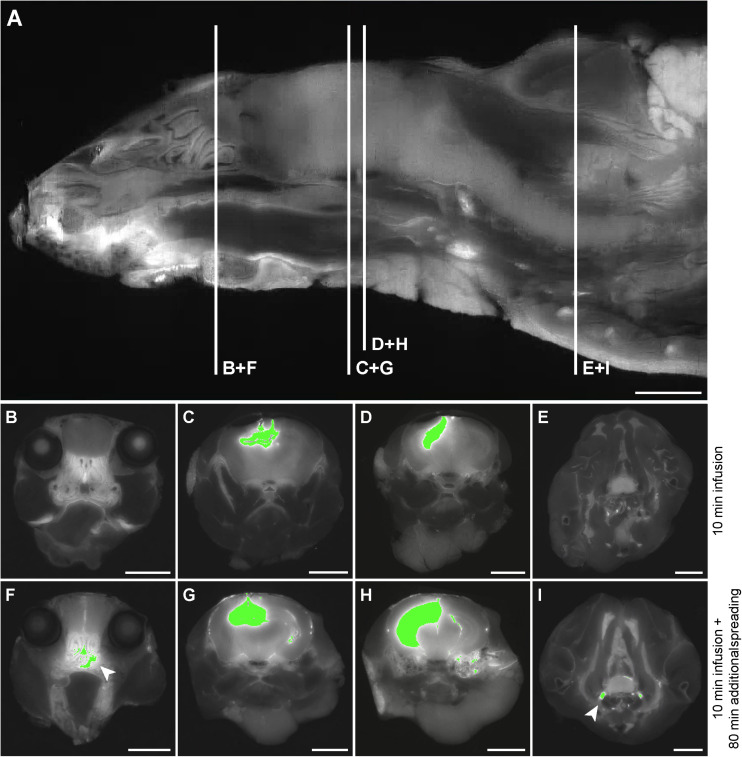
Detail images of tracer dispersion to different anatomical structures. **(A)** A sagittal view of the imaged volume of the mouse. The vertical lines indicate the positions of relevant anatomical structures as shown in the axial views of the mouse in **(B–I)**. Panels **(B–E)** demonstrate segmentation of the fluorescein labeled tracer above the fixed intensity threshold in a mouse sacrificed directly after infusion, while **(F–I)** show this for a mouse that was sacrificed after an additional spreading period of 80 min. **(B)** and **(F)** indicate tracer presence in the nasal turbinates, **(C)** and **(G)** demonstrate this for the infusion site in the CA3 region of the hippocampus, **(D)** and **(H)** for the hippocampus caudal to the infusion site, and **(E)** and **(I)** for the spinal nerves. The arrows indicate tracer dispersion to the nasal turbinates and spinal nerves in **(F)** and **(I)**, respectively. Scale bar represents 3500 μm.

Based on the 3D reconstructions of the tracer distribution as shown in Figure 1, we observed that the two fluorescently labeled dextrans dispersed both in the rostral and in the caudal direction from the infusion site. To get more insight in the spreading into different anatomical structures, we visualized and assessed the tracer dispersion through the central nervous system in the individual 2D images. Due to the limited spatial resolution in the images, small anatomical structures such as the subarachnoid space and cisterns could not be precisely delineated, which precludes the quantitative measurement of tracer signal. The presence of each tracer was therefore scored qualitatively in a number of structures, including adjacent brain regions to the hippocampus, different CSF compartments, and the spinal cord. Table 1 shows the proportion of mice that showed the presence of the two fluorescent dextrans in the different anatomical regions. We observed the presence of tracer in a high proportion of animals in the interpeduncular cistern, and lateral and third ventricles in both experimental groups. This finding suggests that the tracers distributed over the hippocampus and entered the bordering CSF compartments. Once in this compartment, fluorescent dextrans further dispersed through the brain via the subarachnoid spaces and fourth ventricle. Eventually, both tracers ended up in the nasal turbinates and spinal nerves from where they may leave the central nervous system. In Figure 2, detailed images showing the presence of the fluorescein labeled tracers in the two different experimental groups are depicted. Figure 2A demonstrates a sagittal view of a mouse and indicates the positions of representative detail images of the nasal turbinates (Figures 2B,F), infusion site in the hippocampus (Figures 2C,G), dispersion in the hippocampus caudal to the infusion site (Figures 2D,H), and spinal nerves (Figures 2E,I). In Supplementary Figure 3, the dispersion to and through these structures is shown in a sagittal view of a mouse sacrificed after the additional spreading period of 80 min after infusion. A sequential images series from cranial to caudal of a mouse head and thorax is shown in Supplementary Video 1.

**TABLE 1:** Proportion of mice that showed tracer presence in different anatomical structures.

	Fluorescein labeled dextran		Texas Red labeled dextran	
Anatomical structure	10 min infusion (*n* = 3)	10 min infusion + 80 min additional spreading (*n* = 7) *p* = 0.057	10 min infusion (*n* = 3)	10 min infusion + 80 min additional spreading (*n* = 7) *p ≤ 0.05
Nasal turbinates	0.67	1.00	0.33	1.00
Olfactory bulb	0.00	0.57	0.00	0.14
Cortex	0.33	0.43	0.67	0.86
Hippocampus	1.00	1.00	1.00	1.00
Striatum	0.00	0.00	0.67	0.86
Subarachnoid space (cerebrum)	0.33	0.57	0.33	0.86
Lateral ventricle	1.00	0.86	1.00	1.00
Third ventricle	0.67	0.86	1.00	1.00
Fourth ventricle	0.33	0.71	0.14	0.86
Interpeduncular cistern	1.00	0.71	1.00	0.86
Subarachnoid space (cerebellum)	0.33	0.71	0.67	1.00
Spinal cord	0.33	0.86	0.33	1.00
Spinal nerves	0.00	0.86	0.33	1.00

*Fluorescein labeled and Texas Red labeled tracer presence was scored in 13 different anatomical regions, as seen from rostral to caudal. Shown are the proportions of tracer presence for the subset of mice that were sacrificed directly after infusion of the tracer and the group of mice sacrificed after an additional spreading period of 80 min after infusion, referred to as “10 min infusion” and “10 min infusion + 80 min additional spreading,” respectively. To estimate the effect size of the experimental procedure on the dispersion of the fluorescein and Texas Red labeled dextrans, a GEE model was used.*

**FIGURE 3:**
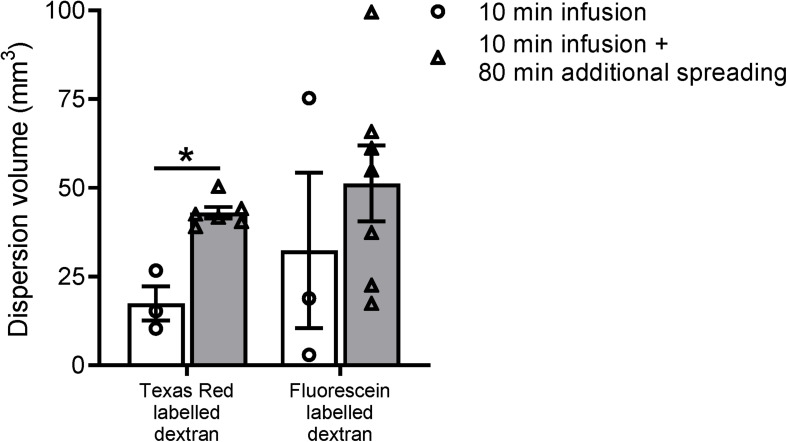
Volume measurements of the tracer dispersion. The dispersion volume was significantly increased for the low molecular weight dextran labeled with Texas Red. For the high molecular weight fluorescein labeled dextran, the dispersion volume was much more variable and did not reach statistical significance. Data represent *n* = 3 for the subset of animals that were sacrificed directly after the infusion period of 10 min, and *n* = 6 or *n* = 7 for the group of mice that were sacrificed after an additional spreading period of 80 min after infusion. Values are mean ± SEM. ^∗^*p* ≤ 0.05 (Mann–Whitney *U* test).

The extent of dispersion during the additional spreading period within the brain and surrounding structures was analyzed by a GEE model for each fluorescent dextran. This revealed an OR of 2.77 and 95% CI of 0.97–7.87 for the fluorescein labeled tracer, with a *p*-value of 0.057. For the Texas Red labeled tracer, the model showed an OR of 5.06 and 95% CI of 1.36–18.82. The corresponding *p*-value of this dextran was 0.015, which indicates strong association between the presence of the Texas Red tracer and additional spreading period that followed infusion.

### Quantification of the Tracer Dispersion Volume

To further study the distribution of the two fluorescent dextrans from the hippocampus in mice, we quantified the total dispersion volume of the individual tracers in the different experimental groups. Even though we used a relatively low infusion rate, it is inevitable that the infusion itself leads to dispersion of the tracer mixture. The spreading of the tracers in the subset of mice sacrificed directly after infusion is therefore expected to be dominated by the infusion of the tracer solution rather than the endogenous mechanisms. Figure 3 shows the dispersion volumes of the two different fluorescently labeled dextrans in both experimental groups. To put these volumes in perspective, the dispersion volumes in the subset of mice that were sacrificed directly after infusion occupy 4–8% of the total mouse brain volume.

Next, we compared the dispersion volumes between the two time points for the individual tracers. We observed that this volume substantially increased after the additional dispersion period of 80 min for Texas Red labeled dextran only. For this tracer, we found a significant 2.5-fold increase in volume after the additional spreading period (*p* ≤ 0.05). The volume of the fluorescein labeled tracer did not significantly change.

## Discussion

In this study, we have examined clearance pathways for solutes from the hippocampus in mice. A custom-built 3D imaging cryomicrotome was used to visualize and quantify the distribution pattern of different fluorescently labeled dextrans. The two tracers distributed over the hippocampus, entered adjacent brain structures, and finally left the brain both in the direction of the nose and the spinal cord. Furthermore, using two time points, we untangled the contribution of experimentally induced tracer spreading from endogenous mechanisms. These data map the removal pathway for solutes and waste products via the ISF and CSF compartment, and may aid in a better understanding of the exact clearance routes from the mouse brain.

Injection of tracer molecules and contrast agents in the brain parenchyma or CSF compartment is a frequently used technique to study the fluid dynamics within the interstitium and the exchange between the CSF and the ISF. These studies require the injection of tracer molecules into the brain, which may affect endogenous processes of tracer dispersion. Previous work from our group and others showed that in rats intrathecal infusion leads to an increase in ICP at infusion rates of 3.0 μL/min or higher (Yang et al., 2013; Bedussi et al., 2017b). In mice, an infusion rate of 0.34 μL/min already slightly elevates the ICP when injected in the cisterna magna (Bedussi et al., 2017a). In the current study, we reduced the infusion rate even further to 0.1 μL/min, in order to minimize changes in ICP. In addition to this low pump rate, we also scaled down the infused tracer solution quantity to 1.0 μL. This quantity is analogous to a volume of 1.0 mm^3^, which may seem a small amount. However, knowing that the extracellular space occupies approximately 20% of the total brain volume, it actually replaces the ISF of 5.0 mm^3^ of brain tissue (Hrabetova et al., 2018). The study by Ma et al. (2005) showed average volumes of 453.2 and 25.7 mm^3^ of the whole brain and hippocampus, respectively, in the same strain of mice used in our study. These numbers demonstrate that the impact of the infusion of even a relatively small quantity of tracer solution in the rodent brain is substantial. Therefore, in order to reveal the tracer distribution due to the infusion itself, we included a subset of mice that was sacrificed directly after infusion. The inclusion of this experimental group allowed us to untangle the contribution of experimentally induced dispersion from dispersion through physiological mechanisms by comparison of distribution volumes with the other group of mice sacrificed after an additional spreading period. Even though we thus minimized both the infusion rate and infused tracer solution quantity, the data from this experimental group show that tracers already distributed over a substantial volume in the hippocampus during the infusion period.

Many studies investigating the fluid transport in the rodent brain rely on the *post mortem* imaging of *in vivo* infused tracer (Iliff et al., 2012; Kress et al., 2014; Albargothy et al., 2018; Berliner et al., 2019). This, in combination with immunohistochemistry, enables a detailed visualization of the tracer distribution ranging from the cellular level up to the whole brain. However, the process of fixation that is often used to preserve the tissue may cause artifacts that could influence the interpretation of the data. A study by Mestre et al. (2018) showed that during perfusion fixation of the animal with 4% paraformaldehyde, the size of the perivascular space changed dramatically, leading to a redistribution of the tracer. This unintended spreading may also develop in brains that are preserved by immersion fixation, as different fixative solutions can cause either shrinkage or swelling of the tissue (McFadden et al., 2019). In addition, one other recent study reported a remarkable discrepancy in the spreading pattern of intrathecally injected tracers between *in vivo* and *ex vivo* measurements in mice. In anesthetized animals, tracer spread was limited to the perivascular spaces of the larger blood vessels on the surface of the brain, whereas immediately after death, tracers rapidly entered these spaces along the penetrating vessels in the brain parenchyma (Ma et al., 2019b). This phenomenon may be explained by a widening of the perivascular spaces as a result of the sudden drop in blood pressure that occurs upon death. These findings, together, indicate that the distribution of fluorescent tracers in histological sections may not accurately reflect the dispersion in living mice.

To avoid these *post mortem* artifacts, studies on the glymphatic system have used *in vivo* imaging techniques as magnetic resonance imaging (MRI) and two-photon microscopy (Iliff et al., 2012, 2013a,b; Kress et al., 2014). These imaging modalities permit the dynamic measurement and visualization of fluid transport in the rodent brain. Besides this noteworthy feature, MRI provides imaging of the entire brain and has therefore become one of the most valuable tools for evaluating CSF–ISF exchange *in vivo*. However, despite the advancements that have recently been made, this technique still has limited spatial and temporal resolution to assess perivascular flow (Mestre et al., 2018). Two-photon laser scanning microscopy overcomes this lack of resolution and therefore allows the detailed imaging of perivascular spaces. However, this technique is only capable of visualizing these spaces in small, superficial areas of the brain cortex, due to its small field of view and restricted penetration depth (Iliff et al., 2013b; Kress et al., 2014).

The custom-built 3D imaging cryomicrotome may overcome a number of these limitations. This device enables sectioning and high-resolution imaging of large tissue samples such as the human heart, as well as whole mice and rats because of its ability to slice through bone tissue. In the current study, this latter characteristic allowed for the detailed visualization of the fluorescent tracers throughout the entire mouse head and thorax. Since this approach does not require the dissection of the brain, all connections within the CSF compartment, including its cisterns and subarachnoid space, remained intact. This revealed important drainage pathways from the brain via these spaces both to the spinal cord and to the cribriform plate that will be easily missed when only examining tracer dispersion in the dissected brain tissue. Another advantage of preserving the animal as a whole is that this shortens the time period between death and tissue fixation, thereby presumably reducing the *post mortem* artifacts of tracer dispersion as described by Mestre et al. (2018) and Ma et al. (2019b).

This technique, furthermore, permits the visualization of multiple fluorophores. As a result, a number of fluorescent probes and other detectable molecules of different sizes can be examined in the same experiment. Because of this property, the imaging cryomicrotome enables the assessment of convective and diffusive transport of tracer molecules in the brain interstitium. In this study, we observed that in the group of mice sacrificed after an additional spreading period after infusion, both fluorescent tracers distributed to various anatomical structures. However, our finding that the extent to which the tracers dispersed through the brain and surrounding structures was larger for the Texas Red labeled tracer suggests an enhanced dispersion of this smaller tracer through the central nervous system. In addition, quantification of the tracer dispersion volume revealed that the actual distribution area only increased significantly after the additional spreading period for this same small, Texas Red labeled tracer. These findings may therefore indicate a prominent role for solute dispersion via diffusion in addition to convection, which appears to result mainly from injection. Tracers distributed over the hippocampus and entered adjacent brain structures, such as the cortex and CSF compartment. Once in the CSF compartment, tracers left the brain via the ventricular system, subarachnoid spaces, and cisterns in the directions of the nose and spinal cord. Tracers subsequently left the central nervous system via the cribriform plate and spinal nerves, after which the signal intensity became too low to detect. These data confirm findings of other studies such as Ma et al. (2019a) and Brady et al. (2020), which reinforce the role of CSF as a clearance pathway for waste materials. Particularly solutes for which no transporters are present at the blood–brain barrier, or insoluble aggregates of molecules, may depend on such mechanisms. Exactly how tracers reach the CSF compartment could not be determined. Despite the higher resolution of the imaging cryomicrotome when compared to other imaging modalities, e.g., MRI, the resolution is still too limited to assess whether tracers dispersed via the perivascular spaces to these structures, or simply crossed boundaries between the brain parenchyma and CSF compartment via relatively permeable structures such as the ependyma.

A methodological limitation of using two different fluorophores is the difference in their specific spectral properties. Fluorescein was detected at short-wavelength emission, at which tissue autofluorescence was also observed in particular structures, such as the nose and blood vessels. As a consequence, the differentiation between the true signal of the tracer and background fluorescence required a relatively high threshold. Even though this threshold was carefully chosen, we cannot fully rule out a minor contribution of tissue autofluorescence to the dispersion volume quantification of this tracer in some cases. This may, in turn, explain the higher variation observed in the dispersion volumes of the fluorescein labeled tracer when compared to the Texas Red labeled tracer. This latter tracer showed a higher signal-to-noise ratio; however, segmentation of this more diffusely spread dextran was still determined by an arbitrary threshold. Since these limitations may hinder the quantitative comparison of tracer molecules with different fluorophores, we did not directly compare the dispersion pattern and volume of the two dextran tracers in this study. Another limitation is the small sample size and restricted number of time points in the current work. In future studies, a larger sample size and the addition of later time points may allow a better discrimination of endogenous mechanisms versus experimentally induced tracer dispersion.

## Conclusion

In conclusion, the present study assessed clearance pathways of solutes from the mouse brain by the use of a whole body imaging procedure. This revealed that fluorescent tracers travel through the hippocampus, enter adjacent brain structures, and eventually leave the brain in the direction of the cribriform plate and spinal cord via the CSF compartment. We also observed that even a limited infusion rate and volume of tracers cause experimentally induced dispersion over a substantial brain volume. In the time frame of the current study, only a low molecular weight tracer showed further distribution, implying an important contribution of diffusion for solute transport in the brain. Taken together, these results reveal how solutes leave the hippocampus, which may aid in a better understanding of waste clearance in the healthy and diseased brain.

## Data Availability Statement

The raw data supporting the conclusions of this article will be made available by the authors, without undue reservation.

## Ethics Statement

The animal study was reviewed and approved by the Academic Medical Center Animal Ethics Committee.

## Author Contributions

DN, EV, and EB conceptualized and designed the study. DN, JV, and EB performed the experiments and acquired the data. DN, JD, EV, and EB were involved in the analysis and interpretation of the data. DN and EB wrote the manuscript. DN, JD, JV, EV, and EB revised the manuscript. All authors contributed to the article and approved the submitted version.

## Conflict of Interest

The authors declare that the research was conducted in the absence of any commercial or financial relationships that could be construed as a potential conflict of interest.

## Supplementary Material

Supplementary Figure 1 3D reconstructions of the fluorescein labeled tracer distribution through the body of the individual mice. The polygon meshes delineate regions above the fixed intensity threshold of the fluorescein labeled dextran. Panels **(A–C)** show the dispersion of this tracer in the individual mice that were sacrificed after the infusion period of 10 min, whereas panels **(D–J)** show this for the individual mice sacrificed after the additional spreading period of 80 min that followed the infusion period of 10 min. Scale bar represents 5000 μm.

Supplementary Figure 2 3D reconstructions of the Texas Red labeled tracer distribution through the body of the individual mice. The polygon meshes delineate regions above the fixed intensity threshold of the Texas Red labeled dextran. Panels **(A–C)** show the dispersion of this tracer in the individual mice that were sacrificed after the infusion period of 10 min, whereas panels **(D–I)** show this for the individual mice sacrificed after the additional spreading period of 80 min that followed the infusion period of 10 min. Scale bar represents 5000 μm.

Supplementary Figure 3 Sagittal view of the tracer dispersion to different anatomical structures. Panel **(A)** shows a raw sagittal image of the imaged volume of a mouse that was sacrificed after an additional spreading period of 80 min. Panel **(B)** demonstrates segmentation of the fluorescein labeled dextran above the fixed intensity threshold in this same image. The arrows indicate tracer dispersion to the nasal turbinates and spinal nerves. Scale bar represents 3500 μm.

Supplementary Video 1 Sequential image series from cranial to caudal in a mouse head and thorax. A sequential image series of axial images show the dispersion of the large, fluorescein labeled dextran tracer through the mouse head and thorax after infusion in the hippocampus.

## References

[B1] Albargothy N. J. Johnston D. A. MacGregor-Sharp M. Weller R. O. Verma A. Hawkes C. A. (2018). Convective influx/glymphatic system: tracers injected into the CSF enter and leave the brain along separate periarterial basement membrane pathways. *Acta Neuropathol.* 136 139–152. 10.1007/s00401-018-1862-7 29754206 PMC6015107

[B2] Bedussi B. Almasian M. de Vos J. VanBavel E. Bakker E. N. (2017a). Paravascular spaces at the brain surface: low resistance pathways for cerebrospinal fluid flow. *J. Cereb. Blood Flow Metab.* 38 719–726. 10.1177/0271678x17737984 29039724 PMC5888857

[B3] Bedussi B. van der Wel N. N. de Vos J. van Veen H. Siebes M. VanBavel E. (2017b). Paravascular channels, cisterns, and the subarachnoid space in the rat brain: a single compartment with preferential pathways. *J. Cereb. Blood Flow Metab.* 37 1374–1385. 10.1177/0271678X16655550 27306753 PMC5453458

[B4] Bedussi B. van Lier M. G. Bartstra J. W. de Vos J. Siebes M. VanBavel E. (2015). Clearance from the mouse brain by convection of interstitial fluid towards the ventricular system. *Fluids Barriers CNS* 12:23. 10.1186/s12987-015-0019-5 26435380 PMC4593194

[B5] Berliner J. A. Woodcock T. Najafi E. Hemley S. J. Lam M. Cheng S. (2019). Effect of extradural constriction on CSF flow in rat spinal cord. *Fluids Barriers CNS* 16:7. 10.1186/s12987-019-0127-8 PMC6434898 30909935

[B6] Braak H. Braak E. (1991). Neuropathological stageing of Alzheimer-related changes. *Acta Neuropathol.* 82 239–259. 10.1007/bf00308809 1759558

[B7] Brady M. Rahman A. Combs A. Venkatraman C. Kasper R. T. McQuaid C. (2020). Cerebrospinal fluid drainage kinetics across the cribriform plate are reduced with aging. *Fluids Barriers CNS* 17:71. 10.1186/s12987-020-00233-0 PMC7706057 33256800

[B8] Davis A. A. Leyns C. E. G. Holtzman D. M. (2018). Intercellular spread of protein aggregates in neurodegenerative disease. *Annu. Rev. Cell Dev. Biol.* 34 545–568. 10.1146/annurev-cellbio-100617-062636 30044648 PMC6350082

[B9] Hrabetova S. Cognet L. Rusakov D. A. Nägerl U. V. (2018). Unveiling the extracellular space of the brain: from super-resolved microstructure to *In Vivo* function. *J. Neurosci.* 38 9355–9363. 10.1523/jneurosci.1664-18.2018 30381427 PMC6706003

[B10] Iliff J. J. Lee H. Yu M. Feng T. Logan J. Nedergaard M. (2013a). Brain-wide pathway for waste clearance captured by contrast-enhanced MRI. *J. Clin. Invest.* 123 1299–1309. 10.1172/jci67677 23434588 PMC3582150

[B11] Iliff J. J. Wang M. Liao Y. Plogg B. A. Peng W. Gundersen G. A. (2012). A paravascular pathway facilitates CSF flow through the brain parenchyma and the clearance of interstitial solutes, including amyloid beta. *Sci. Transl. Med.* 4:147ra111. 10.1126/scitranslmed.3003748 22896675 PMC3551275

[B12] Iliff J. J. Wang M. Zeppenfeld D. M. Venkataraman A. Plog B. A. Liao Y. (2013b). Cerebral arterial pulsation drives paravascular CSF-interstitial fluid exchange in the murine brain. *J. Neurosci.* 33 18190–18199. 10.1523/jneurosci.1592-13.2013 24227727 PMC3866416

[B13] Kress B. T. Iliff J. J. Xia M. Wang M. Wei H. S. Zeppenfeld D. (2014). Impairment of paravascular clearance pathways in the aging brain. *Ann. Neurol.* 76 845–861. 10.1002/ana.24271 25204284 PMC4245362

[B14] Ma Q. Decker Y. Müller A. Ineichen B. V. Proulx S. T. (2019a). Clearance of cerebrospinal fluid from the sacral spine through lymphatic vessels. *J. Exp. Med.* 216 2492–2502. 10.1084/jem.20190351 31455602 PMC6829589

[B15] Ma Q. Ineichen B. V. Detmar M. Proulx S. T. (2017). Outflow of cerebrospinal fluid is predominantly through lymphatic vessels and is reduced in aged mice. *Nat. Commun.* 8:1434. 10.1038/s41467-017-01484-6 PMC5681558 29127332

[B16] Ma Q. Ries M. Decker Y. Müller A. Riner C. Bücker A. (2019b). Rapid lymphatic efflux limits cerebrospinal fluid flow to the brain. *Acta Neuropathol.* 137 151–165. 10.1007/s00401-018-1916-x 30306266 PMC6338719

[B17] Ma Y. Hof P. R. Grant S. C. Blackband S. J. Bennett R. Slatest L. (2005). A three-dimensional digital atlas database of the adult C57BL/6J mouse brain by magnetic resonance microscopy. *Neuroscience* 135 1203–1215. 10.1016/j.neuroscience.2005.07.014 16165303

[B18] McFadden W. C. Walsh H. Richter F. Soudant C. Bryce C. H. Hof P. R. (2019). Perfusion fixation in brain banking: a systematic review. *Acta Neuropathol. Commun.* 7:146. 10.1186/s40478-019-0799-y PMC6728946 31488214

[B19] Mestre H. Tithof J. Du T. Song W. Peng W. Sweeney A. M. (2018). Flow of cerebrospinal fluid is driven by arterial pulsations and is reduced in hypertension. *Nat. Commun.* 9:4878. 10.1038/s41467-018-07318-3 PMC6242982 30451853

[B20] Ross C. A. Poirier M. A. (2004). Protein aggregation and neurodegenerative disease. *Nat. Med.* 10(Suppl.) S10–S17. 10.1038/nm1066 15272267

[B21] Selkoe D. J. (2002). Alzheimer’s disease is a synaptic failure. *Science* 298 789–791. 10.1126/science.1074069 12399581

[B22] Soto C. Pritzkow S. (2018). Protein misfolding, aggregation, and conformational strains in neurodegenerative diseases. *Nat. Neurosci.* 21 1332–1340. 10.1038/s41593-018-0235-9 30250260 PMC6432913

[B23] Spaan J. A. ter Wee R. van Teeffelen J. W. Streekstra G. Siebes M. Kolyva C. (2005). Visualisation of intramural coronary vasculature by an imaging cryomicrotome suggests compartmentalisation of myocardial perfusion areas. *Med. Biol. Eng. Comput.* 43 431–435. 10.1007/bf02344722 16255423

[B24] Tarasoff-Conway J. M. Carare R. O. Osorio R. S. Glodzik L. Butler T. Fieremans E. (2015). Clearance systems in the brain-implications for Alzheimer disease. *Nat. Rev. Neurol.* 11 457–470. 10.1038/nrneurol.2015.119 26195256 PMC4694579

[B25] van den Wijngaard J. P. Schwarz J. C. van Horssen P. van Lier M. G. Dobbe J. G. Spaan J. A. (2013). 3D Imaging of vascular networks for biophysical modeling of perfusion distribution within the heart. *J. Biomech.* 46 229–239. 10.1016/j.jbiomech.2012.11.027 23237670

[B26] Yang L. Kress B. T. Weber H. J. Thiyagarajan M. Wang B. Deane R. (2013). Evaluating glymphatic pathway function utilizing clinically relevant intrathecal infusion of CSF tracer. *J. Transl. Med.* 11:107. 10.1186/1479-5876-11-107 23635358 PMC3665671

