# Supplementary figures and images for: Mapping Solute Clearance From the Mouse Hippocampus Using a 3D Imaging Cryomicrotome
Source: Front Neurosci. 2021 Mar 22;15:631325. doi: 10.3389/fnins.2021.631325 (PMC8044999; doi:10.3389/fnins.2021.631325)

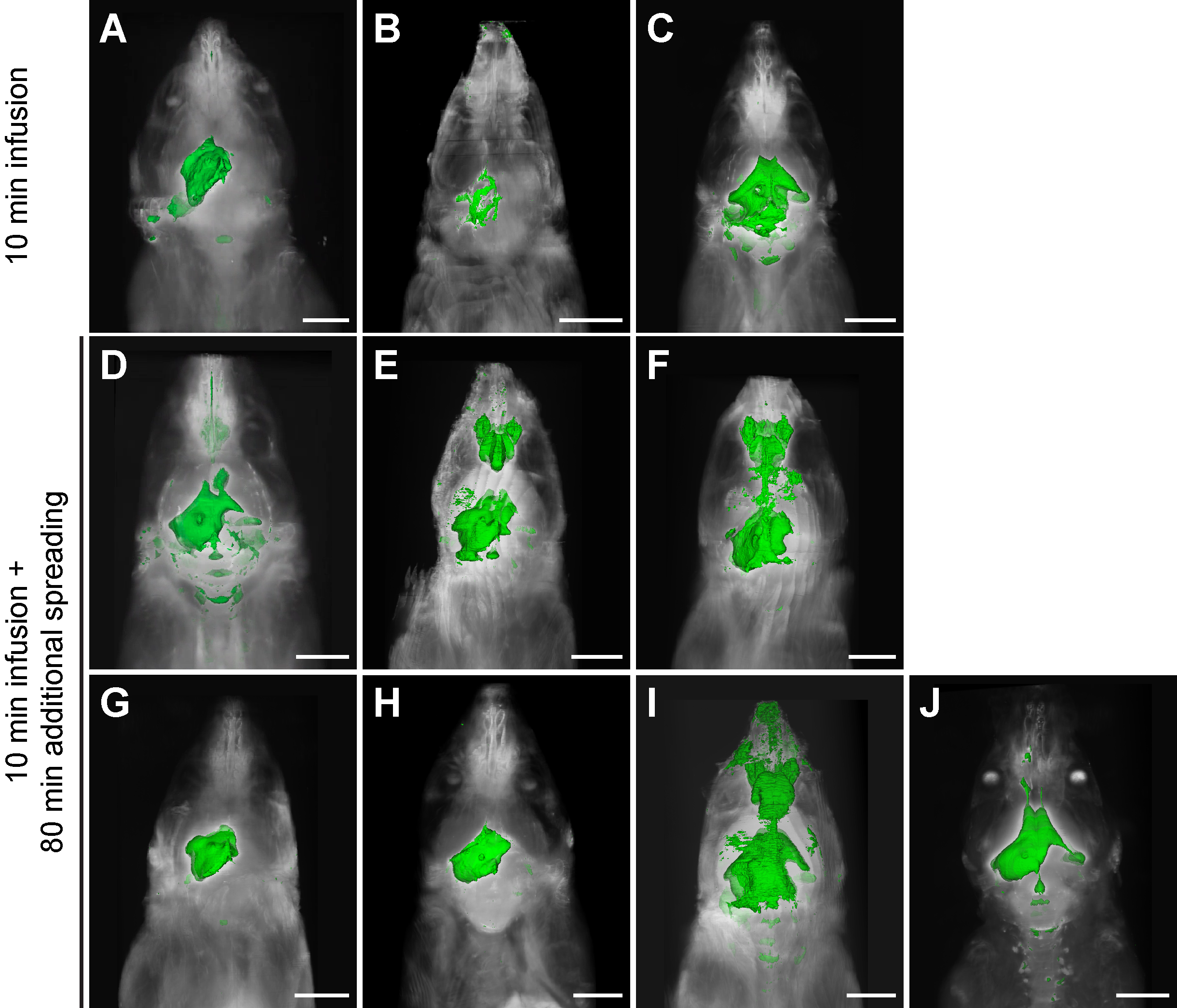

Supplement: Supplementary Figure 1 — 3D reconstructions of the fluorescein labeled tracer distribution through the body of the individual mice. The polygon meshes delineate regions above the fixed intensity threshold of the fluorescein labeled dextran. Panels (A–C) show the dispersion of this tracer in the individual mice that were sacrificed after the infusion period of 10 min, whereas panels (D–J) show this for the individual mice sacrificed after the additional spreading period of 80 min that followed the infusion period of 10 min. Scale bar represents 5000 μm. [file Image_1.TIF]

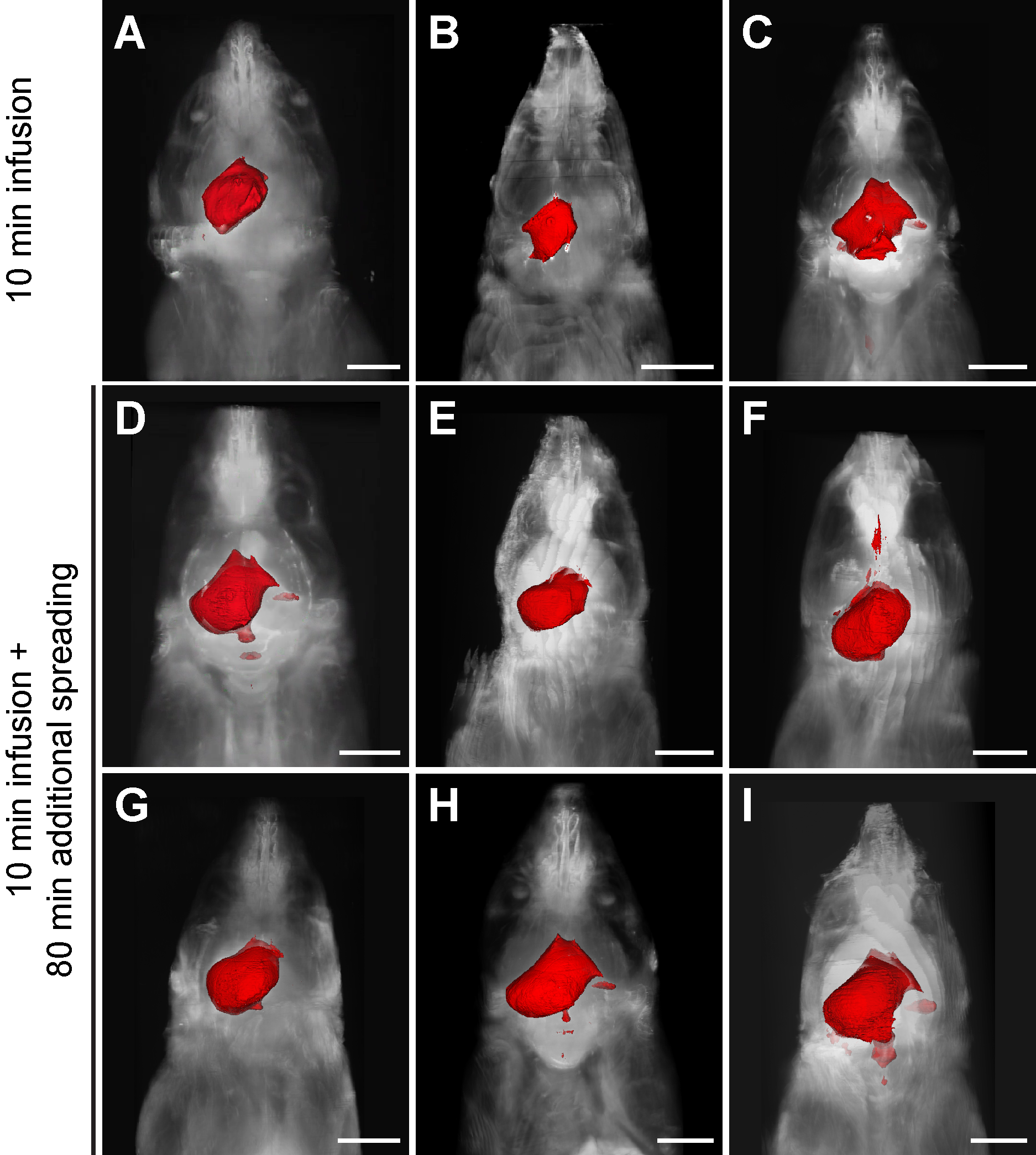

Supplement: Supplementary Figure 2 — 3D reconstructions of the Texas Red labeled tracer distribution through the body of the individual mice. The polygon meshes delineate regions above the fixed intensity threshold of the Texas Red labeled dextran. Panels (A–C) show the dispersion of this tracer in the individual mice that were sacrificed after the infusion period of 10 min, whereas panels (D–I) show this for the individual mice sacrificed after the additional spreading period of 80 min that followed the infusion period of 10 min. Scale bar represents 5000 μm. [file Image_2.TIF]

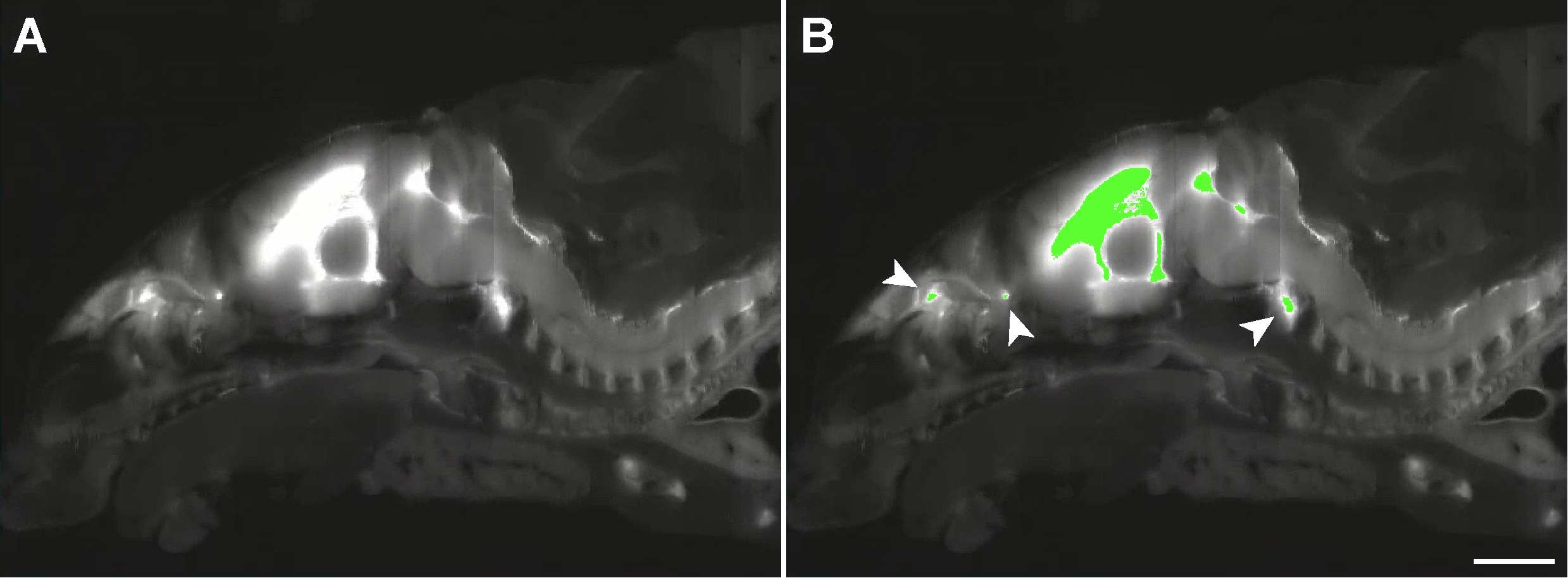

Supplement: Supplementary Figure 3 — Sagittal view of the tracer dispersion to different anatomical structures. Panel (A) shows a raw sagittal image of the imaged volume of a mouse that was sacrificed after an additional spreading period of 80 min. Panel (B) demonstrates segmentation of the fluorescein labeled dextran above the fixed intensity threshold in this same image. The arrows indicate tracer dispersion to the nasal turbinates and spinal nerves. Scale bar represents 3500 μm. [file Image_3.TIF]
